# New insights into acquired endocrine resistance of breast cancer

**DOI:** 10.20517/cdr.2019.13

**Published:** 2019-06-19

**Authors:** Ping Fan, V. Craig Jordan

**Affiliations:** Department of Breast Medical Oncology, The University of Texas MD Anderson Cancer Center, Houston, TX 77030, USA.

**Keywords:** Estrogen receptor α, endocrine resistance, endoplasmic reticulum stress, estrogen-induced apoptosis, breast cancer

## Abstract

The translational research strategy of targeting estrogen receptor α (ERα) positive breast cancer and then using long term anti-hormone adjuvant therapy (5–10 years) has reduced recurrences and mortality. However, resistance continues to occur and improvements are required to build on the success of tamoxifen and aromatase inhibitors (AIs) established over the past 40 years. Further translational research has described the evolution of acquired resistance of breast cancer cell lines to long term estrogen deprivation that parallels clinical experience over years. Additionally, recent reports have identified mutations in the ERα obtained from the recurrences of AI treated patients. These mutations allow the ERα to activate without ligands and auto stimulate metastatic tumor growth. Furthermore, the new biology of estrogen-induced apoptosis in acquired resistant models *in vitro* and *in vivo* has been interrogated and applied to clinical trials. Inflammation and stress are emerging concepts occurring in the process of acquired resistance and estrogen-induced apoptosis with different mechanisms. In this review, we will present progress in the understanding of acquired resistance, focus on stress and inflammatory responses in the development of acquired resistance, and consider approaches to create new treatments to improve the treatment of breast cancer with endocrine resistance.

## INTRODUCTION

Estrogen receptor _α_ (ERα) is a critical nuclear transcription factor to mediate cell proliferation and metabolism through binding to its ligand estrogen (E_2_) in breast cancer. Approximately 70% of breast cancer patients are ER-positive. Thus, targeting ER with tamoxifen or inhibition of E_2_ synthesis via aromatase inhibitors (AIs) are standard treatments for ER-positive breast cancer^[1,2]^. The translational strategy of long term anti-hormone adjuvant therapy targeted to ER has saved millions of women’s life due to effectively control the growth of breast cancer^[3]^. Results from clinical trials demonstrate that the risk of breast cancer relapse is reduced by extending adjuvant tamoxifen therapy from 5 to 10 years^[4,5]^. Consistently, increasing the duration of adjuvant therapy with an AI letrozole to 10 years decreases recurrence rates and contralateral breast cancer^[6]^, whereas Mamounas *et al*.^[7]^ observe that 5 years of letrozole therapy does not significantly prolong disease-free survival compared with placebo to patients with early-stage breast cancer. Unfortunately, acquired resistance to anti-hormone therapy is an inevitable challenge in the clinic after prolonged therapies^[8,9]^. Understanding the mechanisms that underlie this resistance provides an opportunity to develop strategies for overcoming it.

**Figure 1.**
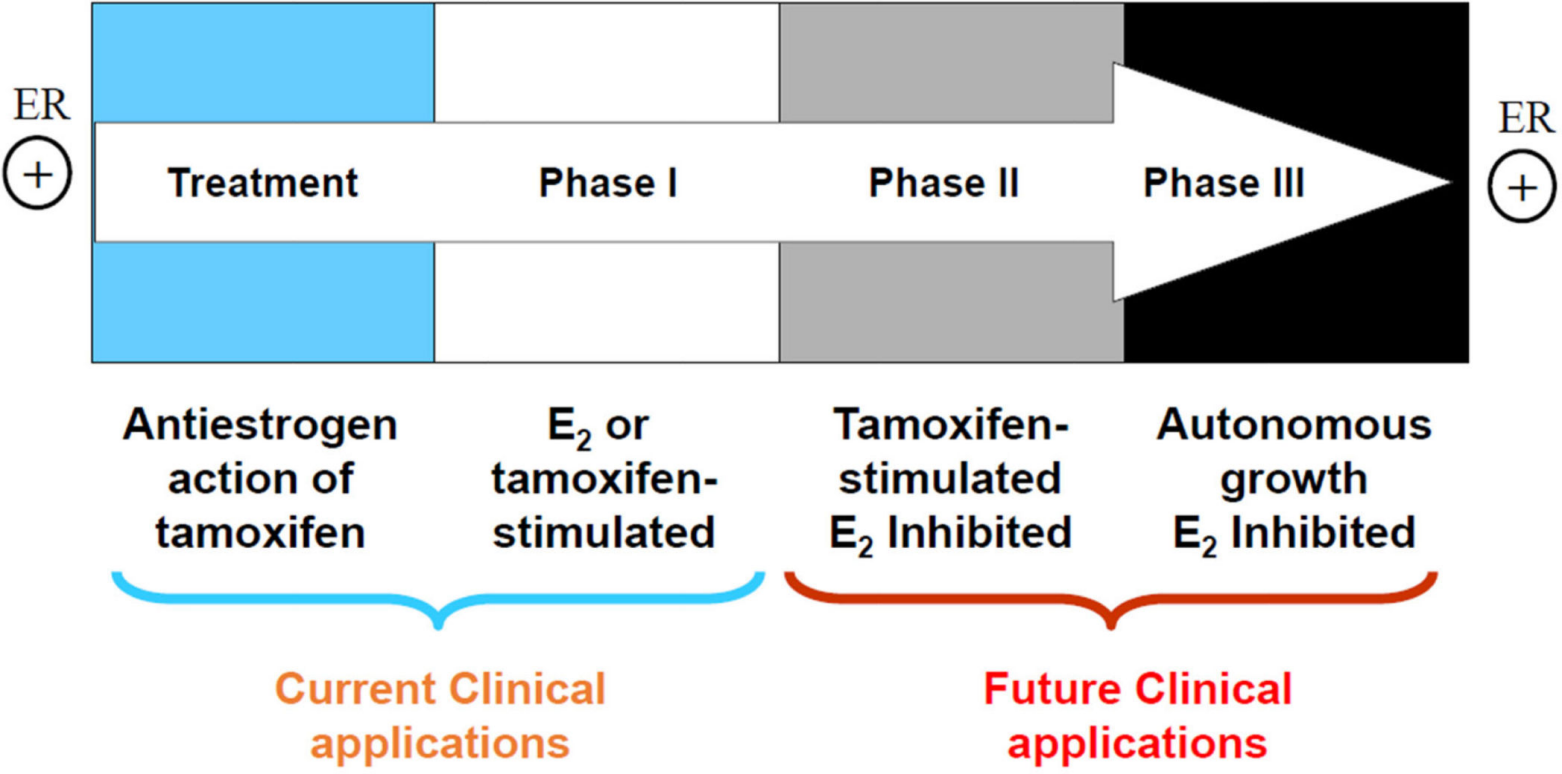
The evolution of resistance to tamoxifen after long term therapy. Phase I acquired resistance develops after a year or two of therapy of estrogen receptor (ER)-positive metastatic breast cancer. Tamoxifen stimulates cell growth, but aromatase inhibitors (AIs) and fulvestrant inhibit cell growth at this stage. Phase II acquired resistance occurs after 5 years of tamoxifen treatment. Tamoxifen continues to stimulate cell growth, but E_2_ induces apoptosis. Eventually, autonomous growth occurs after indefinite tamoxifen therapy for ER-positive breast cancer, which is referred as Phase III acquired resistance. Cells are unresponsive to AIs or fulvestrant, but E_2_ still induces apoptosis^[11^]

There are three types of resistance to selective estrogen receptor modulators (SERMs): metabolic resistance, *de novo* resistance, and acquired resistance^[8]^. Metabolic^[10]^ and *de novo*^[8]^ resistance have been extensively reviewed and will not be considered further. Acquired resistance to tamoxifen has different biological characteristics according to the treatment time on xenografted mouse model [Figure 1]. Initially, tamoxifen acts as E_2_ antagonist in breast cancer to inhibit growth during the successful treatment of breast cancer, but then causes tamoxifen-stimulated breast cancer growth after a year or two of treatment which is a unique form of acquired drug resistance^[3]^. The AIs and fulvestrant can prevent tumor growth in tamoxifen-resistant disease. This phase of drug resistance is referred to as Phase I resistance. In phase II, constant exposure to tamoxifen more than 5 years results in continued tamoxifen-stimulated growth, but E_2_ induces apoptosis at this stage. Eventually, autonomous growth (Phase III) occurs after indefinite treatment that is unresponsive to fulvestrant or AIs, but E_2_ still induces apoptosis at this stage [Figure 1]. The distinct features at different phases of acquired resistance suggest that cell populations are clearly being selected over years of therapy so that those cells can adapt and grow in a stressful environment^[11]^.

Several mechanisms have been proposed to contribute to the acquired endocrine resistance, including activation of growth factor receptor and kinase pathways (e.g., HER-2 and MAPK)^[12–14]^, amplification of transcriptional co-activator proteins (e.g., SRC3)^[15]^, mutations in the ligand-binding domain of ERα^[16]^, and constitutive activation of other inflammation-associated transcription factors such as nuclear factor κB (NF-κB)^[17,18]^. Particularly, inflammation is now considered a hallmark of cancer and plays a key role in all aspects of tumor biology, including initiation, angiogenesis, resistance, and metastasis^[19–21]^. Moreover, inflammatory response suppresses the function of ERα, thereby affecting the response to SERMs^[21]^. Importantly, long term anti-hormone therapy alters the interactions between ERα and other inflammation- and stress-associated transcription factors such as NF-κB and peroxisome proliferator-activated receptor _γ_ (PPAR_γ_)^[18,22,23]^, which ultimately switch the cellular response to E_2_ from proliferation to apoptosis *in vitro*^[24,25]^ and *in vivo*^[26,27]^. This laboratory discovery has been translated into the clinical trial^[28,29]^.

We will review how stressful anti-hormone therapy alters the function of ERα, including its interactions with membrane-associated molecules, mutations, and crosstalk with nuclear inflammation-associated transcription factors, thereby leading to the endocrine resistance. Furthermore, we will focus on how these inflammation-associated transcription factors modulate subsequent E_2_-induced apoptosis and find approaches to improve therapeutic effects of E_2_-induced apoptosis on endocrine resistant breast cancer.

## CROSSTALK BETWEEN GROWTH FACTOR RECEPTORS AND ERα IN ACQUIRED ENDOCRINE RESISTANCE

**Figure 2.**
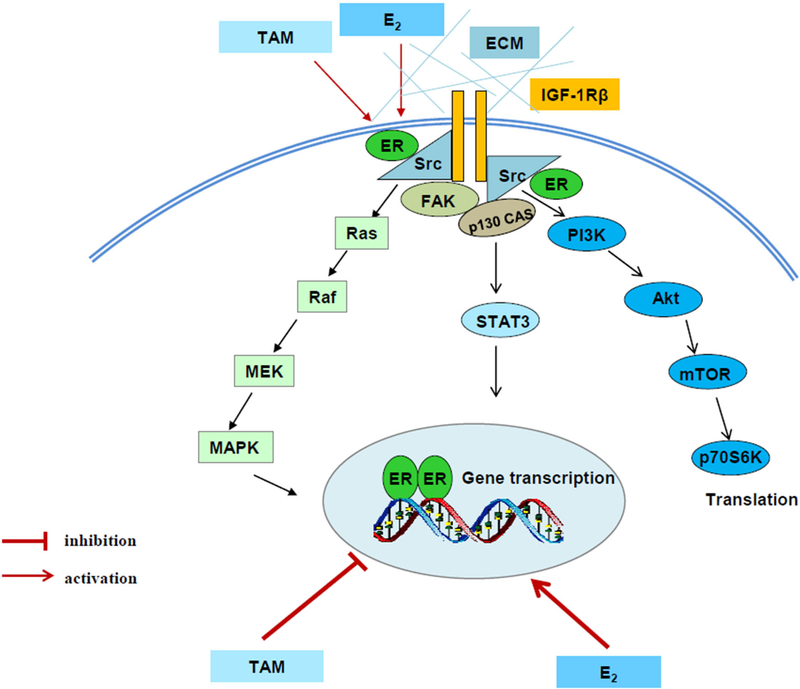
Signal transduction pathways differentially regulated by E_2_ and tamoxifen in tamoxifen-resistant model. E_2_ and tamoxifen (TAM) exert differential functions on nuclear estrogen receptor α (ERα). E_2_ activates classical ER-target genes but TAM acts to block gene activation. Both E_2_ and TAM increase the non-genomic activity of ERα through membrane-associated molecules such as extracellular matrix (ECM), c-Src, insulin-like growth factor-1 receptor (IGF-1R), and focal adhesion kinase (FAK) to enhance downstream signaling cascades, leading to acquired resistance^[32^]

Compelling evidence has demonstrated that several tyrosine kinase receptors, including HER-2^[12,13]^, epithelial growth factor receptor (EGFR)^[30,31]^, and insulin-like growth factor-1 receptor (IGF-1R)^[32,33]^ participate in acquired endocrine resistance in breast cancer. Therefore, the most common strategy for overcoming endocrine resistant breast cancer is inhibition of these tyrosine kinase receptors in breast cancer patients^[34,35]^. In addition to activating downstream signaling pathways such as PI3K/Akt and MAPK, these tyrosine kinase receptors cross talk with ERα and evade the anti-hormone therapy in breast cancer^[12,30–33]^. The interaction between ERα and growth factor receptors results in the redistribution of ERα from nucleus to extra nuclear areas and increasing the non-genomic pathway of ERα, which will further activate PI3K/ Akt and MAPK pathways^[30,32]^. In another aspect, ER_α_ can be phosphorylated by activated MAPK and Akt, resulting in ligand-independent transcriptional activity^[36,37]^. Particularly, aberrant activation of PI3K/Akt by PI3KCA mutations has been implicated in endocrine resistance of breast cancer^[38,39]^. Additionally, more investigations disclose that activity of non-receptor tyrosine kinase c-Src is increased^[40,41]^ and plays a key role in the mediation of interaction between ERα and growth factor receptor in endocrine resistant breast cancer cells^[30]^. Thus, targeting c-Src can overcome acquired endocrine resistance^[30,42]^. Moreover, multiple membrane-associated molecules including focal adhesion molecules, adapter proteins, and growth factor receptors are identified to be activated in SERM-resistant breast cancer cells^[32,43]^ [Figure 2]. These active membrane-associated molecules integrally activate downstream signal pathways and lead to be unresponsive to tamoxifen [Figure 2].

Despite activation of multiple membrane-associated molecules, considerable results implicate that ERα is still a major drive of growth utilized by both E_2_ and SERMs in resistant models *in vivo*^[44,45]^ and *in vitro*^[46]^. In contrast to E_2_ that activates classical ER-target genes, SERMs continue to act as effective antiestrogens to inhibit classical ER-target genes, even at the time of growth stimulation^[32]^. This conclusion is supported by our previous results that growth of tumors by tamoxifen or fulvestrant lacks the induction of E_2_-responsive genes^[47]^. Other groups also reported similar results that tamoxifen suppresses classical ERE-regulated genes during occurrence of acquired resistance *in vitro*^[48]^ and *in vivo*^[49]^. Consistently, expression of IGF-1R is up-regulated by E_2_ through ERα but downregulated by tamoxifen^[32]^, which leads to the loss of IGF-1R in the tamoxifen resistant breast cancer^[50]^. However, phosphorylation of IGF-1R is increased by tamoxifen due to the non-genomic activity of ERα^[32]^. All of these findings suggest that SERMs consistently inhibit classical ERα transcriptional activity regardless of being SERM sensitive or resistant breast cancer cells.

In contrast, ERα tethering pathway such as activator protein-1 (AP-1) family members are activated in tamoxifen resistant breast cancer^[51–54]^. Apart from interaction with other transcription factors, stress-related kinases such as c-Jun NH2-terminus kinase (JNK) has been documented to activate AP-1 proteins through phosphorylation^[53–55]^. In line with these results, JNK and p38 have been found to promote acquired resistance in breast cancer^[56]^. Notably, our findings demonstrate that both JNK and Akt are commonly regulated by IGF-1R in SERM resistant breast cancer cells^[57]^. All together, these factors including growth factor receptors, stress-associated kinases, and AP-1 family members are activated after anti-hormone therapy, which contribute to SERM-resistance in ER-positive breast cancer.

## ERα MUTATION OCCURRENCE IN ACQUIRED RESISTANT BREAST CANCER

ERα continues to be expressed in the majority of cases with acquired endocrine resistance. However, the function of ERα has been altered after long term endocrine therapies. Recent genetic studies have shown that high frequency of *ERα* gene (*ESR1*) mutations (around 20%) occurs in acquired resistant tumors^[58]^, mainly in AI resistant tumors. In contrast, it is extremely rare to find ERα mutations in primary breast cancer^[16,58,59]^, including those matched primary tumors from patients in which ERα mutations are found after progress of endocrine resistance^[58]^. These results suggest that selective pressures from endocrine therapies are critical for the acquisition of *ESR1* mutations^[16,58,60]^. Remarkably, several *ESR1* point mutations identified in acquired resistant breast cancer occur in ER ligand binding domain (LBD)^[16,58–63]^, just a few amino acids within or near the helix 12 region of the LBD. This region has function for undergoing conformational changes during ERα activation^[16,58–64]^. Amino acid 351 allele mutation was first found in MCF-7 xenografted tumors after long term tamoxifen treatment^[64,65]^. The majority of other mutation sites are detected in metastatic breast cancer tumors or cell lines after long term endocrine therapies^[58,61,62,66–68]^. Based on these findings, Tyr537 and Asp538 are the hot spots of *ESR1* mutations^[58,61,62,66–68]^. These single allele mutations do not affect the dimerization of ERα, but they continuously increase the transcriptional activity of ERα^[67,68]^, which result in the loss of response to tamoxifen and fulvestrant. Of note, ERα mutations are also enriched in PI3KCA mutant tumors and most of these point mutations are ERα phosphorylation sites^[69]^. Tyr537 is a unique site phosphorylated by c-Src^[70]^, which is implicated in hormone binding, dimerization, and hormone-dependent transcriptional activity. Other mutation sites are phosphorylated at serine residues through RAS/MAPK^[36]^ or downstream signal of growth factor receptors^[71–73]^. It remains unclear whether ERα mutation is related with over activation of kinases after acquired endocrine resistance. Recently, Mao *et al*.^[74]^ reported that Y537S mutation constitutively increases the unfolded protein response (UPR) with high expression of XBP1 and Bip/GRP78, which are associated with tamoxifen resistance. However, ER biomodulator, BHPI further elicits UPR in breast cancer cells with ERα mutations^[74]^. This persistent activation of UPR converts cell responses from protection to death, leading to completely inhibit proliferation of breast cancer cells with ERα mutations^[74,75]^. Moreover, some novel antiestrogens or selective estrogen receptor down-regulators are developed to overcome acquired resistance caused by ERα mutations^[76–78]^. All of these results highlight the importance and functional consequence of ERα mutations and provide an important resource for studying endocrine resistance of breast cancer.

## ALTERATION OF INTERACTION BETWEEN ERα AND INFLAMMATION-ASSOCIATED TRANSCRIPTION FACTORS AFTER ACQUIRED RESISTANCE

In addition to the critical role in female reproduction, E_2_ directly modulates lipid metabolism and the function of mitochondria, thereby influencing adipocyte differentiation and energy homeostasis^[79–82]^. Thus, E_2_ deficiency caused by menopause or anti-hormone therapies results in metabolic stress, demonstrating fat redistribution and insulin resistance^[80–83]^. Specifically, fatty acid and cholesterol metabolism are increased after endocrine therapy or menopause in breast cancer cells, along with abnormal activation of cytokines locally and distally^[83,84]^. Both inflammatory factors and lipid metabolism regulators [such as PPARγ, sterol regulatory element-binding protein 1(SREBP1), and CCAAT/enhancer binding protein β (C/EBP β)] have been identified to result in acquired resistance in breast cancer^[84–88]^. In particular, PPARγ is a master adipocyte modulator to affect the lipid and energy metabolism^[89,90]^, which function is closely related with the levels of E_2_^[23,86,91]^. Many observations have demonstrated that a bidirectional crosstalk exists between ERα and PPARγ in the regulation of proliferation, differentiation, metabolism, and inflammation in breast cancer^[92–95]^. Additionally, NF-κB is another key transcription factor in responsible for inflammation and acquired resistance in ER-positive breast cancer^[96–98]^. Long term endocrine therapy is inclined to create an inflammatory microenvironment in breast cancer^[99]^. It has been reported that cytokines and chemokines released in the inflammatory environment activate NF-κB-associated pathways that desensitize cell response to SERMs^[99]^. Therefore, repression of NF-κB activity can restore sensitivity to ERα antagonists^[100]^. An inverse relationship between ERα and NF-κB has been observed in the development of endocrine resistant breast cancer^[101,102]^. E_2_ has a potential to suppress the activation of NF-κB^[18]^. However, long term anti-hormone therapy alters the function of ERα in the regulation of metabolism and inflammation^[103]^ that results in the constitutive activation of NF-κB^[18]^. Apart from interaction with ERα, the activity of NF-κB is suppressed by C/EBPβ and PPARγ^[18,23,104,105]^. Particularly, trans-suppression of NF-κB by the PPARγ agonist is a major mechanism underlying inhibition of inflammation and acceleration of insulin sensitivity in the clinic^[106,107]^. Furthermore, the inflammatory factor is able to reprogram the motifs on ERα binding sites on chromatin which is closely associated with the endocrine resistance in breast cancer^[108,109]^. These results demonstrate that long term anti-hormone therapy alters the function of ERα and its interaction with inflammation-associated transcription factors that results in the endocrine resistance in breast cancer.

## E_2_-INDUCED APOPTOSIS IN ACQUIRED RESISTANT BREAST CANCER

The primary purpose of anti-hormone therapy is to prevent E_2_ from binding to ERα (SERMs) or inhibit synthesis of E_2_ (AIs) in ER-positive breast cancer, thereby blocking the proliferation of breast cancer cells. Paradoxically, administration of physiological concentrations of E_2_ can induce apoptosis in acquired resistant breast cancer *in vitro*^[24,25]^ and *in vivo*^[26,27]^. This scientific discovery has been used in the clinical trial for the treatment of aromatase inhibitor resistant breast cancer patients with 30% of benefit^[29]^. This rationale is also been used to interpret why E_2_ alone hormone replacement therapy (HRT) can reduce the incidence of breast cancer in hysterectomized postmenopausal women^[110]^. Our further clinically relevant findings disclose that the synthetic progestin medroxyprogesterone acetate for the classical HRT in combination with E_2_ has glucocorticoid activity that is able to block E_2_-induced apoptosis^[111]^, implicating that inflammation and stress are involved in the apoptosis induced by E_2_^[112–115]^. In support with this view, it has been reported that long term anti-hormone therapy is stressful for breast cancer, along with activation of multiple stress- and inflammation-associated transcription factors and pathways^[18,23,57,116–119]^. Furthermore, E_2_ treatment widely activates stress responses including endoplasmic reticulum stress, oxidative stress, and inflammatory stress in long term estrogen deprivation (LTED) breast cancer cells^[117,118]^. Among these stress responses, the endoplasmic reticulum is a critical regulatory site for conveying signals between the nucleus and cytoplasm to induce apoptosis^[18,118]^. Three sensors of endoplasmic reticulum stress are activated but perform different functions after E_2_ treatment. One of these sensors, protein kinase RNA-like endoplasmic reticulum kinase (PERK) is responsible for homeostasis of unfolded proteins and plays a critical role in E_2_-induced apoptosis^[57,118]^. The other two sensors, inositol-requiring protein 1 alpha (IRE1α) and ATF-6, mainly mediate endoplasmic reticulum-associated degradation of phospholipids^[57]^. Differential functions of the endoplasmic reticulum stress sensors suggest that abnormal protein folding and lipid metabolism occur after exposure to E_2_ in LTED breast cancer cells^[23,57]^.

**Figure 3.**
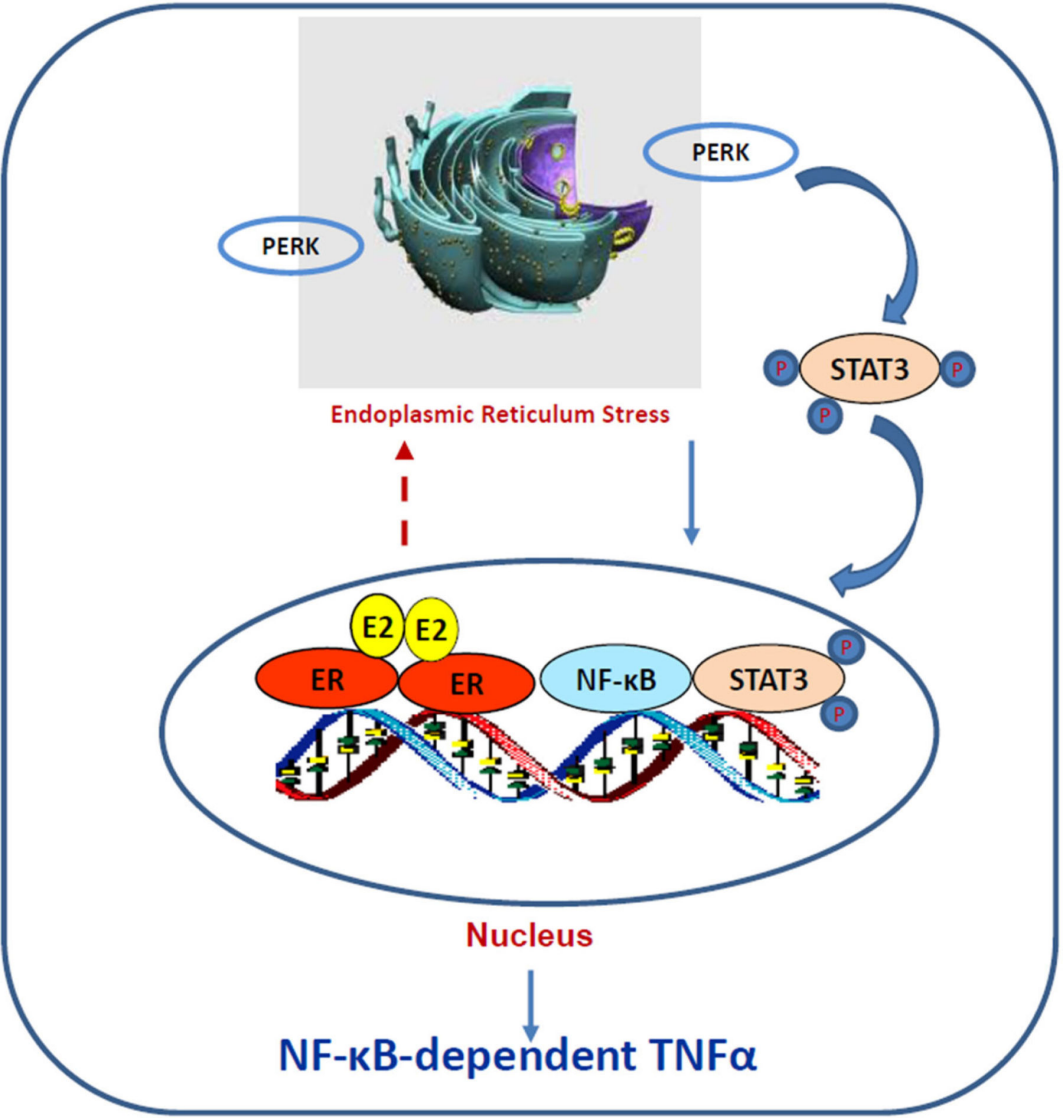
The protein kinase RNA-like endoplasmic reticulum kinase (PERK)/nuclear factor κB (NF-κB)/tumor necrosis factor _α_ (TNF_α_) axis is activated by E_2_ to induce apoptosis in long term estrogen deprivation breast cancer cells. E_2_ activates nuclear estrogen receptor _α_ (ER_α_) and accumulates unfolded proteins in the endoplasmic reticulum, which activates PERK in response to the misfolded proteins. This stress kinase phosphorylates signal transducer and activator of transcription 3 (STAT3) and increases its DNA-binding activity. Subsequently, activated STAT3 promotes NF-κB DNA binding and induction of TNF_α_ expression^[22^]

**Figure 4.**
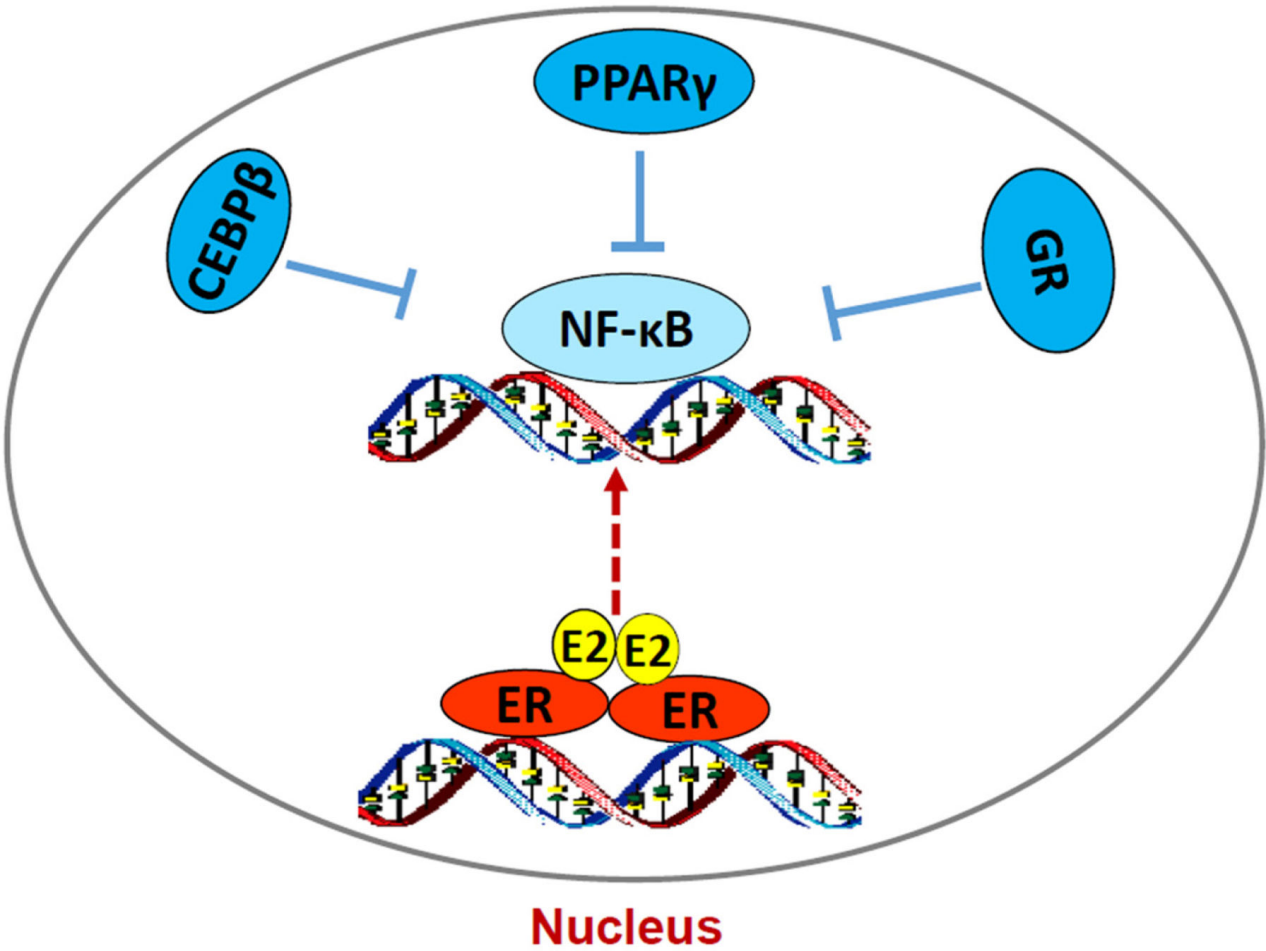
Regulation of nuclear factor κB (NF-κB) DNA-binding activity by other transcription factors in acquired resistant breast cancer cells. E_2_/estrogen receptor _α_ (ER_α_) activates endoplasmic reticulum stress and subsequently increases NF-κB DNA-binding activity. However, lipid metabolism-associated transcription factors CCAAT/enhancer binding protein _β_ (C/EBP _β_) and peroxisome proliferator-activated receptor _γ_ (PPAR_γ_) and inflammation modulator GR all suppress the DNA-binding activity of NF-κB, thereby inhibition of E_2-_induced apoptosis in long term estrogen deprivation breast cancer cells

How ERα triggers stress responses remain unclear. Although E_2_-induced stress utilizes c-Src tyrosine kinase^[46,118]^, a well-known molecule to mediate non-genomic pathway of ERα^[30,32]^, it is confirmed that nuclear ERα mediates E_2_-induced stress and apoptosis, but not non-genomic effects of ERα^[118]^. The fact that PERK activation is crucial for E_2_-induced apoptosis suggests an accumulation of unfolded proteins in the endoplasmic reticulum after E_2_ treatment^[57,118]^. Most likely, E_2_ increases the expression of some short half-life nuclear proteins such as AP-1 family member c-Fos^[120]^, which is increased by E_2_ and leads to rapid increase the misfolded protein in the endoplasmic reticulum^[121]^. Our recent findings demonstrate that PERK kinase increases DNA-binding activity of signal transducer and activator of transcription 3 which promotes NF-κB translocation to nucleus and activation of NF-κB-dependent tumor necrosis factor α (TNFα)^[18,22]^. The PERK/ NF-κB/TNFα axis is identified as the key drive to induce apoptosis after E_2_ treatment of LTED breast cancer cells^[18,22]^ [Figure 3]. In addition of regulating apoptosis, NF-κB is a critical molecule to mediate metabolism, stress, inflammation, and proliferation, depending on the context of cells^[122]^. It also has a close crosstalk with other transcription factors to regulate inflammatory responses^[102]^. For example, nuclear receptor PPARγ suppresses the function of NF-κB in a variety of cells^[103,105]^. Our results show that PPARγ agonist suppresses the NF-κB DNA-binding activity and blocks E_2_-induced apoptosis in LTED breast cancer cells^[23]^. In contrast, antagonist of PPARγ inhibits breast cancer cell growth^[23,123,124]^ and increases E_2_-induced apoptosis via regulation of oxidative stress and NF-κB-dependent TNFα expression^[23]^. Furthermore, the mechanism underlying glucocorticoids blockade of E_2_-induced apoptosis is also mediated by selective suppression of NF-κB DNA-binding activity and subsequent inhibition of TNFα expression in LTED breast cancer cells^[121]^. Thus, nuclear activity of NF-κB can be modulated by multiple other transcription factors to affect therapeutic effects of E_2_-induced apoptosis in anti-hormone resistant breast cancer [Figure 4]. These findings also imply that biological function of ERα is affected by several other transcription factors, depending on the ligands present in the nucleus.

## CONCLUSION

In summary, long term anti-hormone therapy is a stress pressure for ER-positive breast cancer cells that selects cell populations for the adaptation to the treatment^[11]^. During adaptation, multiple stress- and inflammation-associated transcription factors and pathways are activated and participate in the promotion of acquired endocrine resistance. Simultaneously, these stress and inflammatory responses create a microenvironment facilitating E_2_-induced apoptosis in the acquired resistant breast cancer cells. Remarkably, ERα is the initial target for the endocrine therapy whereas it is also the initial site to trigger apoptosis. Although ERα continues to be expressed, the interactions between ERα and stress- and inflammation-associated transcription factors such as NF-κB and PPARγ are altered when resistance occurs^[18,23]^. The ultimate effects of stress and inflammation can either promote proliferation or induce apoptosis, depending on the context of tumor cells^[96,125,126]^. Generally, persistent stress makes cell susceptible to apoptosis^[127]^. Therefore, many novel compounds are designed to manipulate stress responses for the therapy of diseases^[74,127]^. Collectively, the discovery of E_2_-induced apoptosis not only has clinical relevance to treat aromatase inhibitor-resistant breast cancer and reduce breast cancer incidence in postmenopausal women^[29,110]^, but also a general principal has emerged to understand sex steroid-induced apoptosis in long-term androgen deprived prostate cancer^[128]^. Ongoing strategic studies in our laboratory are addressing the mechanisms underlying sex steroid-induced apoptosis in a range of models of normal and cancer cells.
